# A co-adaptive duality-aware framework for biomedical relation extraction

**DOI:** 10.1093/bioinformatics/btad301

**Published:** 2023-05-23

**Authors:** Weiyan Zhang, Chuang Chen, Jiacheng Wang, Jingping Liu, Tong Ruan

**Affiliations:** School of Information Science and Engineering, East China University of Science and Technology, 130 Meilong Road, Shanghai 200237, China; School of Information Science and Engineering, East China University of Science and Technology, 130 Meilong Road, Shanghai 200237, China; School of Information Science and Engineering, East China University of Science and Technology, 130 Meilong Road, Shanghai 200237, China; School of Information Science and Engineering, East China University of Science and Technology, 130 Meilong Road, Shanghai 200237, China; School of Information Science and Engineering, East China University of Science and Technology, 130 Meilong Road, Shanghai 200237, China

## Abstract

**Motivation:**

Biomedical relation extraction is a vital task for electronic health record mining and biomedical knowledge base construction. Previous work often adopts pipeline methods or joint methods to extract subject, relation, and object while ignoring the interaction of subject–object entity pair and relation within the triplet structure. However, we observe that entity pair and relation within a triplet are highly related, which motivates us to build a framework to extract triplets that can capture the rich interactions among the elements in a triplet.

**Results:**

We propose a novel co-adaptive biomedical relation extraction framework based on a duality-aware mechanism. This framework is designed as a bidirectional extraction structure that fully takes interdependence into account in the duality-aware extraction process of subject–object entity pair and relation. Based on the framework, we design a co-adaptive training strategy and a co-adaptive tuning algorithm as collaborative optimization methods between modules to promote better mining framework performance gain. The experiments on two public datasets show that our method achieves the best *F*1 among all state-of-the-art baselines and provides strong performance gain on complex scenarios of various overlapping patterns, multiple triplets, and cross-sentence triplets.

**Availability and implementation:**

Code is available at https://github.com/11101028/CADA-BioRE.

## 1 Introduction

**Figure 1.**
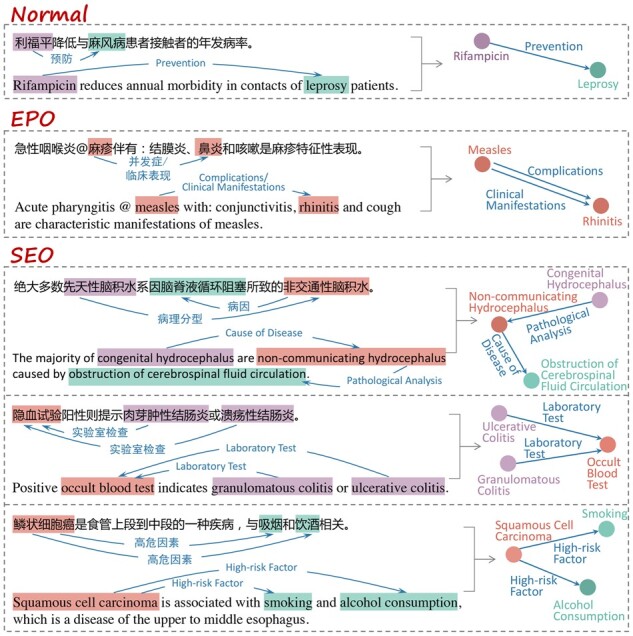
Examples of normal, EPO, and SEO.

The Relation Extraction (RE) task refers to extracting structured information from natural language texts, which has many downstream applications, e.g. automatic knowledge base construction (Li et al. 2019), information retrieval (Luo et al. 2022b) and recommendation (Zhang et al. 2022a). In the biomedical domain, the broad application of electronic health record (EHR) systems causes an exponential increase of EHRs that cover the phenotypes description and patient treatment (Zhao et al. 2022). Biomedical relation extraction (BioRE) aims to automatically identify medical terminologies and mines the interrelation between diseases and drugs/symptoms (Zhao et al. 2021). It is a significant text mining and knowledge discovery task for exploring research in clinical therapy, pathology, drug discovery, etc. (Luo et al. 2022c). For instance, in Fig. 1, given the sentence “利福平在降低与麻风病患者接触的人员年发病率方面有效˚(Rifampicin reduces annual morbidity in contacts of leprosy patients.),” the entity recognition subtask first identifies the subject “利福平 (Rifampicin)” and the object “麻风病 (leprosy),” then the purpose of the relation classification (RC) subtask is to recognize the relation “预防 (prevention)” to the entity pair, and finally the triplet [利福平 (Rifampicin),” “预防 (prevention),” “麻风病 (leprosy)”] is extracted.

**Figure 2.**
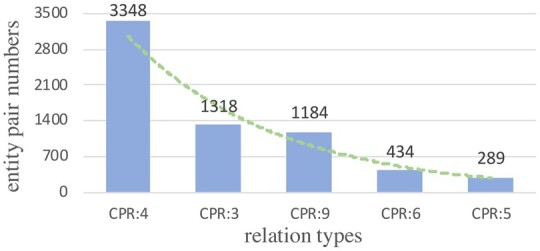
The number of entity pairs corresponding to different relation types in the English public BioRE dataset (i.e. ChemProt).

**Figure 3.**
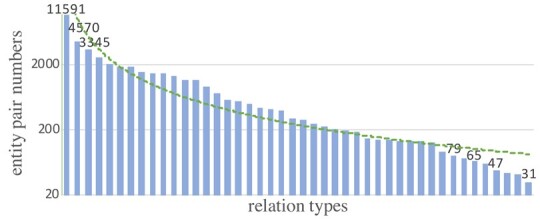
The number of entity pairs corresponding to different relation types in the Chinese public BioRE dataset (i.e. CMeIE).

In this article, we mainly focus on the BioRE task of extracting (subject, relation, object) triplets for a given biomedical text. Compared to the general news domain, BioRE requires much broader and more specialized domain knowledge. In other words, BioRE has more domain-specific difficulties. Specifically, (i) most of the benchmark datasets for BioRE task are imbalanced, e.g. the ChemProt dataset whose more than 79.2% entity pairs are negative instances (i.e. no relations). Furthermore, there is a data imbalance between entity pairs and relation types, i.e. a long-tail problem for relation types. As shown in Figs 2 and 3, regardless of the number of relation types, the BioRE datasets have the long-tail phenomenon, and the difference is only in the degree of severity. It is a prominent challenge to model the interdependence between entity pair and relation within a triplet. (ii) Compared to the general RE task, BioRE has more complex problems of nested entities and overlapping relations due to the unique text structure of biomedical texts and the professional recording habits of EHRs. As shown in Fig. 1, relation overlap is divided into entity pair overlap (EPO) and single entity overlap (SEO) (Luo et al. 2022a). In addition, cross-sentence triplets and multiple relations in a single sentence are more significant difficulties in BioRE (Zhao et al. 2021). (iii) Compared to English texts, Chinese BioRE is more challenging since Chinese EHRs are recorded without explicit word delimiters (Cai et al. 2021). The motivation for studying the BioRE task is accurately identifying biomedical concepts and knowledge, which can provide effective decision support for patient treatment.

Existing methods on the BioRE, in general, can be roughly divided into pipeline methods and joint methods. The former transforms the BioRE task into two subtasks, i.e. entity recognition (ER) (Fei et al. 2021) and RC (An et al. 2022). They first employ a sequence labeling model to extract all potential subjects and objects from the text and then adopt a classifier to identify the reasonable relation type from the extracted subject–object entity pairs (Zhong and Chen 2021). However, pipeline methods ignore additional joint features between entities and relations, which causes two issues: (i) error propagation, as the mistakes in early steps cannot be corrected in later steps (Zheng et al. 2021) and (ii) insufficient interaction of entity pairs and relations within the triplet structure (Shang et al. 2022). To alleviate the above issues, they design joint methods to identify entities and extract relations between them simultaneously. Nevertheless, these joint methods focus on various joint algorithms but ignore that they are essentially based on separate label spaces (Ye et al. 2022).

**Figure 4.**
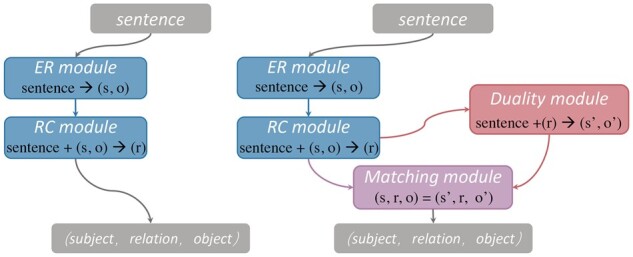
Illustration of the differences between the proposed CADA framework and the existing pipeline-based framework. Left: pipeline-based framework employs an ER module to extract entity, then an RC module to identify relation for obtaining triplets. Right: CADA framework leverages a duality module for inverse extracting triplets and a matching module to correct errors generated in early steps 39.

**Figure 5.**
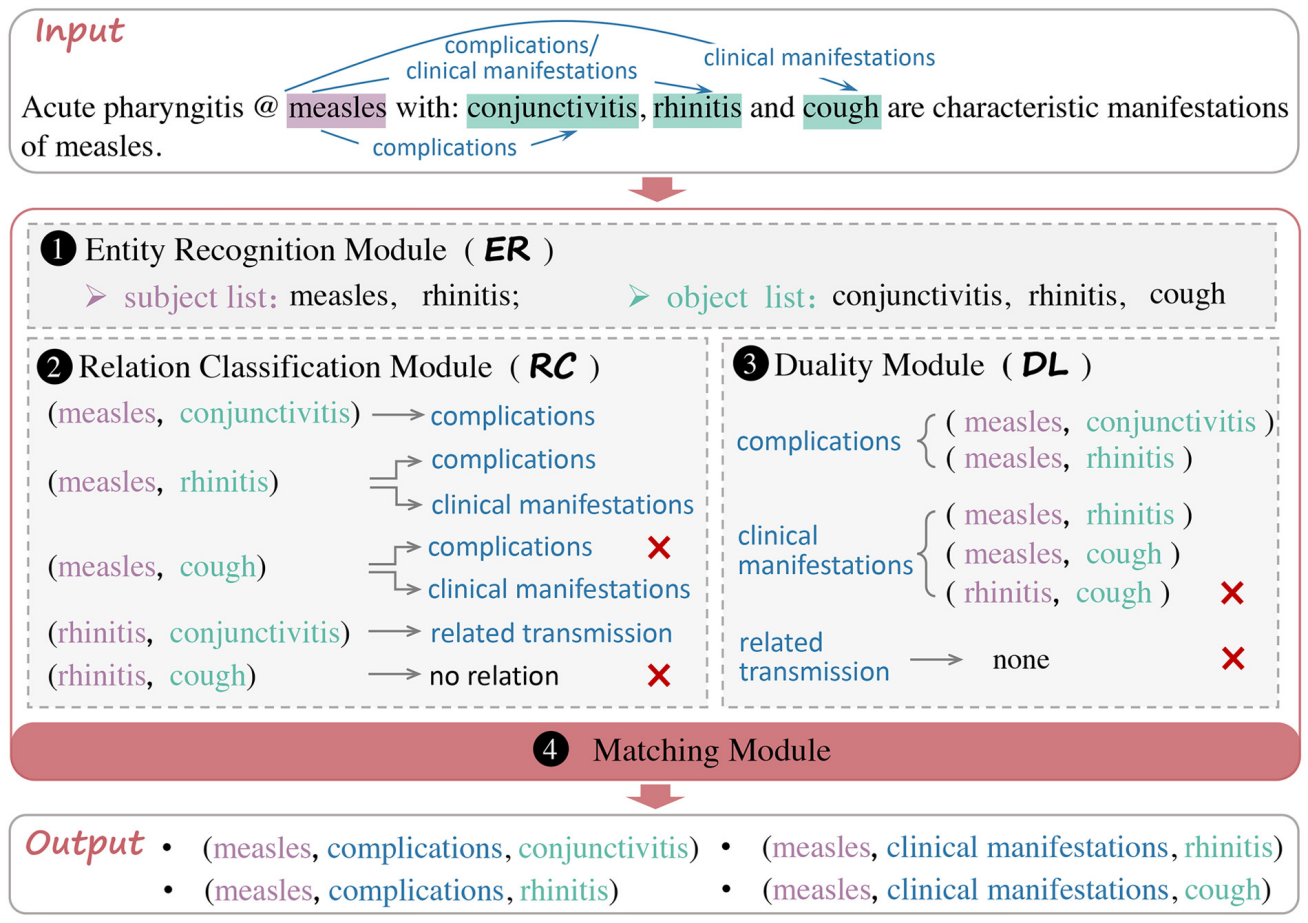
An illustrative example of BioRE subtasks.

To address the above difficulties, in this article, we propose a Co-Adaptive Duality-Aware framework (CADA) for BioRE. As shown in Fig. 4, compared to the conventional pipeline-based RE process, our approach enables bidirectional extraction triplets by adding two steps, i.e. duality module (DL) and matching module. The core idea of CADA is to introduce the duality-aware task into the pipeline BioRE paradigm, to improve the correlation between entity pairs and relations, correct the errors in the early steps and alleviate the limitation of data imbalance through duality mechanism self-checking of entity pairs. Specifically, in this method, we first utilize the span-based entity model to identify the subject–object entity pairs, i.e. ER module. Then, we adopt a classification model combined with entity marker techniques to classify the subject–object entity pairs, i.e. RC module. Afterward, we design the duality task into CADA, i.e. DL, which employs relation types by prompt learning techniques to obtain subject–object entity pairs. Finally, we design a matching module to filter error triplets by inter-checking subject–object entity pairs. In addition, we design a co-adaptive training strategy and a co-adaptive tuning algorithm (CA algorithm), which aim to deliver a consistent and valid performance gain for the overall CADA framework. An illustrative example of BioRE subtasks using the CADA is shown in Fig. 5.

Our contributions are summarized as follows:

- To the best of our knowledge, we are the first to fully consider the interaction of subject–object entity pairs and relations in the BioRE task. We further propose a novel extraction framework based on the duality-aware mechanism (DA).
- We design a co-adaptive training strategy and a CA algorithm that aim to co-optimize all modules in the framework, to bring a consistent and valid performance gain for CADA.
- Our proposed method achieves outstanding performance gains even in complex scenarios involving various overlapping patterns, multiple triplets, and cross-sentence triplets. The experimental results unequivocally demonstrate that the CADA outperforms state-of-the-art (SoTA) BioRE methods.

## 2 Materials and methods

In this section, we first outline task definitions and solutions. Then, we introduce four modules of CADA: ER module, RC module, duality module, and matching module. Finally, we present the training and inference strategies of the CADA framework.

### 2.1 Overview

In this subsection, we first define the task of BioRE and then outline the framework of solution.

#### 2.1.1 Task formulation

In this article, our BioRE task is to extract sentence-level entities and relations in fixed-domain. Given a biomedical text $C=\{c_{1},\ldots ,c_{n}\}$ with *n* tokens, we aim to output a set of triplets t∈T: where *s*, *r*, *o*, and E refer to the subject, relation, object, and the set of entities $e=\{c_{h},c_{h+1},\ldots ,c_{t}\}$, *h* and *t* are the begin and the end of *e*, respectively. Note that *s* and *o* could be an entity extracted from *C*, and $S=\{s_{1},\ldots ,s_{i}\}$ denotes *i* subject in a *C*, and $O=\{o_{1},\ldots ,o_{j}\}$ denotes *j* objects in the same *C*. Therefore, there are *N* different combinations of *e* to form subject–object entity pairs (s,o), where N=i×j. Besides, $R=\{r_{1},\ldots ,r_{l}\}$ denotes *l* predefined relations. For instance, given “Positive occult blood test indicates granulomatous colitis or ulcerative colitis.”, the BioRE task ideal output is (“granulomatous colitis, laboratory test, occult blood test”) and (“ulcerative colitis, laboratory test, occult blood test”).


(1) T={(s,r,o)|s,o∈E,r∈R},

#### 2.1.2 Framework

**Figure 6.**
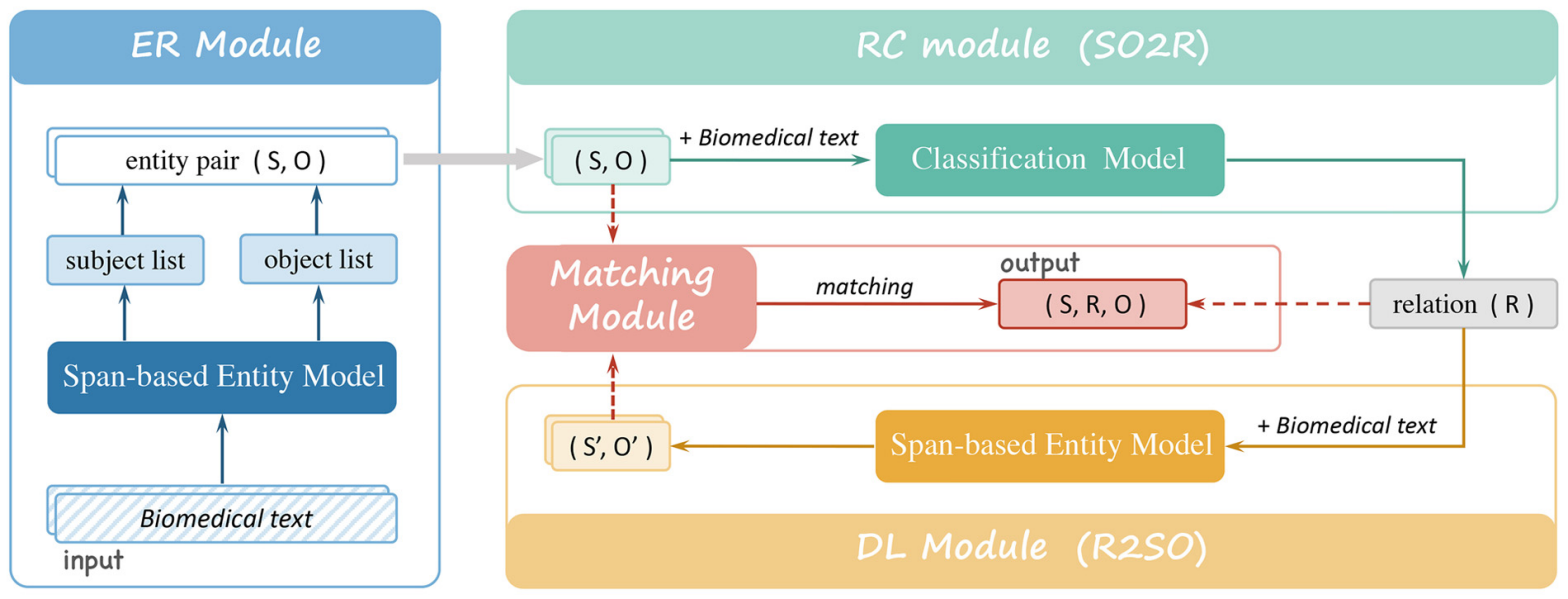
The framework of BioRE via co-adaptive duality-aware.

To deal with the BioRE task, we propose a novel framework named CADA, as shown in Fig. 6. Hence, given a biomedical text *C*, our solution to extract a triplet (s,r,o) in two directions: (i) from (s,o) to *r* (denoted as SO2R) and (ii) from *r* to (s,o) (denoted as R2SO). In other words, we exploit the DA to model the BioRE task in two directions, aiming to correct errors in earlier steps by inter-checking the twice results of entity boundary recognition.

**SO2R.** This direction is to first obtain (s,o) and then identify *r* with the help of *s* and *o*, respectively. Furthermore, identifying *r* is modeled as a classification problem since the *r* is derived from a predefined *R*. As shown in Fig. 6, the modeling modules for this direction are ER and RC.

**R2SO.** This direction is to first obtain *r* and then extract (s,o) with the help of *r*, respectively. In fact, *r* is a benefit for extracting (s,o). This direction is modeled as the DL module shown in Fig. 6.

**Matching.** Ideally, the triplets extracted by modeling two directions are the same. Nevertheless, error propagation makes the triplets extracted in the two directions often differ. Therefore, we design the matching module to inter-checking and filter error triplets.

### 2.2 Entity recognition module

The ER module aims to take biomedical texts *C* as input and obtain entity mentions of subject *s* and object *o*, not needing to obtain entity types. To exploit the model’s ability, we transform NER into an entity boundary detection task (Su et al. 2022). To acquire entity *e*, we adopt a standard span-based model (e.g. global pointer network denote as GP) following prior work (Zhong and Chen 2021; Su et al. 2022). The GP’s core idea is to employ a global matrix to record the probabilities of all spans, which effectively alleviates particularly prominent in BioRE, i.e. nested entity extraction and overlapping RE.

Formally, given the input *C*, we first utilize a context-sensitive pre-trained language model (pre-trained language model (PLM), e.g., BERT) to output context-sensitive representations for each input token *c*. Hence, we have where [CLS] and [SEP] are the special tokens in the BERT model. Then, we define the matrix as $M\in R^{n\times n}$, and *n* is the number of tokens in the input *C*. Each element $M_{h,t}(1\leq h,h\leq t)$ in the matrix refers to the probability of the span starting at the *h*-th token and ending at the *t*-th token, i.e. where $W_{1},W_{2}$ and $b_{1},b_{2}$ are the trainable weights, $R_{h},R_{t}$ are the rotary matrices (Su et al. 2021) used to add relative position information into $v_{h}$ and $v_{t}$, respectively. Thus, the span detection is modeled as a binary problem, where the input is $M_{h,t}$, and the output is true or false. After getting the *S* and *O* from a biomedical text, the *s* and *o* are combined in full permutation to form the subject–object entity pairs (s,o), removing the (s,o) when *s* and *o* are the same entity.


(2) $$v_{C}^{er}=\mathrm{PLM}\left(\left[\mathrm{CLS}][c_{1},c_{2},\ldots ,c_{n}][\mathrm{SEP}\right]\right),$$

(3) $$M_{h,t}={\left(W_{1}v_{h}+b_{1}\right)}^{T}R_{h}^{T}R_{t}\left(W_{2}v_{t}+b_{2}\right),$$

### 2.3 Relation classification module

The RC module aims to take the subject–object pair (s,o) as input and predicts a relation *r* from predefined R or none. Given the output of ER module, we process every pair of the (s,o) set in each text *C* independently in the RC module.

Specifically, we employ the Entity Marker Technique (Zhang et al. 2017) to highlight the (s,o) in text *C*. Namely, we mark the position of *s* by inserting special tokens [*s*] and [/s] at the starting and ending, respectively. Note that the *o* follows the same process, using special tokens [*o*] and [/o]. Subsequently, we take the marked text with insert four special tokens as input to the context-sensitive PLM and obtain the text representation from the output. That is: where $v_{k}^{rc}(k=1,\ldots ,n+4)$. Then, we adopt the representation of the special token $v_{s}^{rc}$ and $v_{o}^{rc}$ as the contextual embedding vectors of *s* and *o*, respectively. Finally, concatenate $v_{s}^{rc}$ and $v_{o}^{rc}$ as the input of the relation classifier (e.g. MLP) to get a *r* or none.


(4) $$v_{C}^{rc}=\left[v_{1},v_{2},\ldots ,v_{n+4}\right]=\mathrm{PLM}\left(\left[c_{1},c_{2},\ldots ,c_{n+4}\right]\right),$$

### 2.4 Duality module

**Figure 7.**
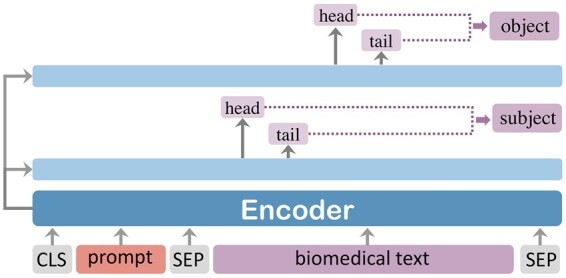
The architecture of the R2SO mode.l

The DL module aims to take the relation $r_{p}^{rc}\in R^{rc}$ obtained by the RC module as input and predicts subject set $S^{\prime }$ and object set $O^{\prime }$. Thus, the model employed is called R2SO, as shown in Fig. 7. In other words, the inputs and outputs of the R2SO task are the opposite of the SO2R task, forming duality tasks. The core idea is to design the DL module to correct the errors in early modules.

Concretely, given a text *C* and a relation $r_{p}^{rc}$, we aim at extracting the corresponding multiple subject–object pairs $\left(s^{\prime },o^{\prime }\right)$. Similar to the ER module, the output of subject–object pair (s,o) also employs the span-based entity model. Inspired by prompt learning (Li and Liang 2021), we design prompt templates to concatenate *r* and *C* as input to the span-based entity model. To fully utilize the semantic information of labels and combine the experimental results of Section 3.4.2, we adopt the relation type, subject type, and object type as the prompt template, which is used as prefixes to the text. That is: where *P* is the prompt template and $v_{C}^{dl}$ is the output vector of PLM. $r_{\mathrm{type}}$, $s_{\mathrm{type}}$, and $o_{\mathrm{type}}$ are separated by the special tokens [/s1], [/s2], and [/s3], which are the sequences of type names for *r*, *s*, and *o*. Finally, we obtain each text’s $s^{\prime }$ and $o^{\prime }$ sets through the DL module.


(5) $$\begin{array}{c} P=\left[/s1\right]\left\{r_{\mathrm{type}}\right\}\left[/s2\right]\left\{s_{\mathrm{type}}\right\}\left[/s3\right]\left\{o_{\mathrm{type}}\right\}, \end{array}$$

(6) $$v_{C}^{dl}=\mathrm{PLM}\left(\left[\mathrm{CLS}\right]P\left[\mathrm{SEP}\right]\left[c_{1},c_{2},\ldots ,c_{n}\right]\left[\mathrm{SEP}\right]\right),$$

### 2.5 Matching module

The matching module aims to take the entity pairs $\left(s^{\prime },o^{\prime }\right)$ obtained by the DL module and (s,o) obtained by the ER module, matching them to get final triplet results. Specifically, two filtering rules are designed for matching triplets. Namely, (i) based on the top-1 relation type score $\gamma_{RC}$ by RC module, we design a threshold $\gamma_{r}$. When $\gamma_{RC}$ is higher than $\gamma_{r}$, the current triplet (s,r,o) is retained regardless of DL module results. (ii) Based on the (s,r,o), we retrieve whether *s* and *o* are contained in the $S^{\prime }$ and $O^{\prime }$ sets, respectively. We keep the current triplet (s,r,o) if they both do. Otherwise, we delete it. In other words, the DL module acts as a dual self-checking. Finally, the BioRE results are obtained after matching the filtering rules.

### 2.6 Training and inference

To maximize the performance of the CADA, we optimize the framework in two parts: training phase and inference phase. In this subsection, we first introduce the training details and then describe the inference process.

#### 2.6.1 Co-adaptive training strategy

During the training phase, the co-adaptive training strategy is used for the entire CADA framework. The core idea is to get more candidate triplets in ER and RC modules, aiming to prevent to loss of correct triplets, i.e. ensuring high recall. Specifically, make the ER and RC modules train according to high recall and the DL module train according to high *F*1 scores. In addition, we adopt a data enhancement [i.e. rdrop (Wu et al. 2021)] for the DL module. Formally, to learn the parameters of the above modules, we exploit a cross-entropy loss function: where Θ is the parameters to be learned by the model, and Score is a scoring function, i.e. the sigmoid function. H denotes the hidden vector, which is the final output of each model.


(7) L(Θ)=−log Score(H),

Algorithm 1 The Co-adaptive Tuning Algorithm

**Input:**

 ER_inference_threshold$=\{a_{1},a_{2},\ldots ,a_{n}\}\in (0.3,0.6]$,

 RC_inference_threshold$=\{b_{1},b_{2},\ldots ,b_{n}\}\in [0.1,0.5]$,

 DL_inference_threshold$=\{c_{1},c_{2},\ldots ,c_{n}\}\in (0.3,0.6]$,

 matching_threshold$=\{d_{1},d_{2},\ldots ,d_{n}\}\in [0.8,1]$;

**Output:** $T^{*}$ : the CADA framework with the best combination of thresholds;

1: **for all** ER_inference_threshold **do**

2:  **for all** RC_inference_threshold **do**

3:   compute the result of the highest recall;

4:  **end for**

5: **end for**

6: **for all** DL_inference_threshold **do**

7:  **for all** matching_threshold **do**

8:   compute the result of the highest *F*1 score;

9:  **end for**

10: **end for**

11: **return**$T^{*}$

#### 2.6.2 Co-adaptive tuning algorithm

During the inference phase, the CA algorithm is employed for the entire CADA framework. The core idea is to ensure high *F*1-score results to provide a stronger filtering performance in final matching. The CA algorithm is a heuristic algorithm that utilizes our collective experience to effectively narrow down the search space and provide an optimal feasible solution within the defined range, to deliver a consistent and valid performance gain for the framework. Specifically, we exploit four parameters to control the inference process of CADA, including inference thresholds of three core modules and a matching threshold, i.e. ER inference threshold, RC inference threshold, DL inference threshold, and matching threshold. Each of the four thresholds mentioned above is assigned a value range based on prior experience. Algorithm 1 demonstrates the procedure of the co-adaptive algorithm (CA) based on the heuristic. First, ER inference threshold and RC inference threshold are appropriately lower, and their combination is determined by recall. The purpose is to allow more candidate triplets to enter the DL module by properly expanding the prediction range. Afterward, by adjusting the DL inference threshold and matching threshold, the threshold combination is determined by the highest *F*1-score to moderate the filtering strength and avoid mistakenly deleting the correct samples.

## 3 Experiments

In this section, we describe the experimental setup. Then, we conduct extensive experiments to evaluate the effectiveness of the proposed CADA framework and analyze its properties. Furthermore, we also design experiments to validate performance gain on complex scenarios. Finally, we perform some detailed analysis.

### 3.1 Experimental setup

#### 3.1.1 Datasets

To evaluate CADA, we used the Chinese BioRE dataset and the English BioRE dataset, respectively. First, we perform our experiments on the Chinese Medical Information Extraction (CMeIE) dataset that comes from Chinese Biomedical Language Understanding Evaluation 2.0 (Zhang et al. 2022b). The detailed statistics of CMeIE are shown in Table 1. As the CMeIE test labels are not released, we cannot count the test set according to the triplet situation. Thus, the experiments on complex scenarios are completed on the validation set. Another, the BioCreative VI ChemProt corpus (https://biocreative.bioinformatics.udel.edu/tasks/biocreative-vi/track-5/) covers chemical-protein relations (Krallinger et al. 2017), the vast majority of them being at the sentence level. The detailed statistics of ChemProt are shown in Table 2. In the dataset, both chemical and protein mentions are pre-annotated.

**Table 1.** Statistics of CMeIE.^a^

Class		Train	Valid	Test
# Entities		11	11	11
# Relations		44	44	44
# Samples		14 339	3585	4482

# Overlapping patterns	Normal	5506	1425	-
	EPO	189	40	-
	SEO	8806	2150	-

# Multiple triplets in a sample	1	5333	1380	-
	2	2932	779	-
	3	1871	433	-
	4	1323	312	-
	≥5	2880	681	-

# Triplets distances	Single-sentence	7524	1900	-
	Cross-sentence	6815	1685	-

a We count CMeIE based on overlapping patterns, noting that a sample can belong to both EPO and SEO. "-" denotes none. Additionally, we consider the number of triplets in a sample and whether triplets is cross-sentence, respectively.

**Table 2.** Statistics of ChemProt.

Class		Train	Valid	Test
#Entities		2	2	2
#Relations		5	5	5

#Entity types	Chemical	13 017	8004	10 810
	Protein	12 752	7567	10 019

# Relation types	CPR: 3	768	550	665
	CPR: 4	2254	1094	1661
	CPR: 5	173	116	195
	CPR: 6	235	199	293
	CPR: 9	727	457	644
	Total	4157	2416	3458

#### 3.1.2 Evaluation metrics

In the following experiments, we first tune our model and run the CA algorithm on the validation set that is separate from the test set, and then make predictions on the test set to determine the final outputs. To evaluate the results of BioRE, we employ three standard evaluation metrics, i.e. precision (*P*), recall (*R*), and strict *F*1 score (*F*1). As the CMeIE test labels are not released, the final evaluation scores are obtained by submitting the entire prediction file to the official website, which requires the (s,r,o) results to be accurately matched. In other words, the extracted triplet can only be considered correct when the triplet elements extracted by the model exactly match the answer.

#### 3.1.3 Compared methods

Since the CMeIE is a Chinese BioRE dataset and ChemProt is an English BioRE dataset, we need to compare the baselines, respectively. We compare our method with SoTA methods as follows on the CMeIE.

- **Pipeline methods.** The typical pipeline extraction methods mainly include the benchmark of **CBLUE** (Zhang et al. 2022b), **PURE** (Zhong and Chen 2021), and **Global Pointer** (Su et al. 2022).
- **Joint methods.** The representative joint extraction methods include **CasRel** (Wei et al. 2020), **HTS-bert** (Yang et al. 2022), **TPlinker** (Wang et al. 2020), **Multi-BERT-wwm** (Zhang et al. 2021), **GPLinker**, **Cas-CLN/Cas-CLN-Syn** (Chang et al. 2022), and **BTCAMS/BTCAMS-Syn** (Chang et al. 2022).

We compare CADA with SoTA methods as follows on the ChemProt.

- **Pipeline methods.** The typical pipeline extraction methods mainly include **Recognition network** (Corbett and Boyle 2018), **SVM+I-ANN** (Mehryary et al. 2018), Majority voting (Peng et al. 2018), **SPINN** (Lim and Kang 2018), **GA-BGRU** (Lu et al. 2019), **CPP-BiLSTM** (Zhang et al. 2019), and **DS-LSTM** (Sun et al. 2019).
- **Joint methods.** The representative joint extraction methods include **CasRel** (Wei et al. 2020), **MRC4BioER** (Sun et al. 2022), **GPLinker** (https://spaces.ac.cn/archives/8888), and **BERT+Gaussian** (Sun et al. 2020).
- **Biomedical PLMs** is to design different pre-training tasks to train domain language models on the biomedical corpus of different scales. The representative biomedical language models include **BERT+DARE** (Papanikolaou and Pierleoni 2020), **BERT+balance bagging** (Papanikolaou and Pierleoni 2020), **NCBI BERT** (Peng et al. 2019), **BioBERT** (Lee et al. 2020), **PubMedBERT** (Gu et al. 2021), and **BioLinkBERT** (Yasunaga et al. 2022).

#### 3.1.4 Parameter setting

We utilize the Chinese version of PLMs [e.g. RoBERTa (https://huggingface.co/clue/roberta_chinese_base)] and the English version of PLMs [e.g. RoBERTa (https://huggingface.co/roberta-base)] released by Huggingface as the text encoder in CMeIE and ChemProt, respectively, which the hidden size is 768. We tune our model on the validation set and use grid search to adjust important hyper-parameters. Specifically, the maximum text length is set to 256, the single input batch size during training in CMeIE is set to 24, the training epoch is set to 10, the Adam optimizer is used for model optimization, the learning rate is set to 4e-5, and the weight decay is set to 0.01. Besides, the single input batch size, epoch, learning rate, and weight decay during training in ChemProt are set to 24, 5, 3e-5, and 0.01, respectively, and the Adam optimizer is used for model optimization. In ER, RC, and DL modules, the drop rate in CMeIE and ChemProt is set to 0.3, 0.4, and 0.3, respectively. The method in this article is completed on a workstation with Ubuntu 20.04.5 LTS, Intel(R) Xeon(R) E5-2678 v3 CPU, GeForce RTX 3090, and 128 GB memory.

### 3.2 Overall results

As mentioned in the introduction, BioRE has two subtasks. To fully verify the effectiveness of CADA, we conduct experiments not only on BioRE task and subtasks, but also on Chinese and English datasets, respectively.

#### 3.2.1 Results of the main experiment

First, we compare CADA for BioRE on Chinse CMeIE. Table 3 shows the results for the different methods. From the table, we conclude that: (i) our method outperforms the competitors on *F*1, demonstrating the effectiveness of CADA. The best performance on BioRE task of CMeIE is 63.13%. (ii) Among all the baselines, the Global Pointer and PURE perform best in pipeline methods, and the BTCAMS-Syn and GPLinker perform best in joint methods. But our method still outperforms pipeline methods by 2.34% and 5.03% on *F*1 and outperforms joint methods by 2.18% and 2.56%, respectively. The reason may be that introducing the DL module into the pipeline method effectively enhances the connection between entity pairs and relations, and adding the matching module corrects the errors in the early steps. (iii) Comparing GPLinker+CADA with GPLinker, adding DL and matching modules behind the joint method increases *F*1 by 1.14%, which shows that introducing the DA also affects joint methods. (iv) Comparing CADA${}^{-}$ and CADA with joint methods shows that a reasonable solution to the drawbacks of pipeline methods can defeat joint methods.

**Table 3.** Main experiment results on the test set of Chinese CMeIE.^a^

Method	Pipeline			Method	Joint		
	*P* (%)	*R* (%)	*F*1 (%)		*P* (%)	*R* (%)	*F*1 (%)
ALBERT-tiny	41.75	32.23	36.38	CasRel${}_{\mathrm{ERNIE}}$	56.78	50.76	53.60
ALBERT-xxlarge	53.96	51.13	52.51	CasRel${}_{\mathrm{BERT}}$	60.61	55.09	57.72
BERT-base	54.28	54.76	54.52	CasRel${}_{\mathrm{BERT}-\mathrm{wwm}}$	60.80	55.02	57.76
BERT-wwm-ext	53.60	54.05	53.82	CasRel${}_{\mathrm{RoBERTa}-\mathrm{wwm}}$	60.45	56.57	58.44
MacBERT-base	54.46	53.98	54.22	HTS-bert	61.16	55.94	58.43
MacBERT-large	57.41	46.00	51.07	TPLinker	63.18	55.85	59.29
MedBERT-base	53.85	53.42	53.63	Multi-BERT-wwm	63.42	56.23	59.30
PCL-MedBERT	54.17	53.75	53.96	Multi-BERT-wwm+FGM	64.67	56.30	60.19
RoBERTa-large	55.21	56.56	55.88	Cas-CLN	65.40	53.90	59.09
RoBERTa-wwm-ext-base	54.43	54.49	54.46	Cas-CLN-Syn	61.09	58.18	59.60
RoBERTa-wwm-ext-large	56.46	56.16	56.31	CAMS	59.92	58.39	59.14
ZEN-base	54.44	53.19	53.81	CAMS-Syn	60.43	58.63	59.52
PURE	55.19	61.33	58.10	BTCAMS	63.96	56.78	60.16
Global Pointer	64.64	57.37	60.79	BTCAMS-Syn	64.51	57.08	60.57
CADA${}^{-}$(our)	63.95	61.92	**62.92**	GPLinker	67.07	55.84	60.95
CADA(our)	**65.44** ^a^	60.98	**63.13**	GPLinker+CADA	69.49	56.12	62.09

CADA${}^{-}$ indicates that the CA algorithm is not used.

a The bold values denote the SoTA results.

Second, we compare CADA for BioRE on English ChemProt. The results are listed in Table 4. From the table, we observe that: (i) our method outperforms all baselines in terms of *P* and *F*1, reaching 78.30% and 77.75%, respectively, which proves the effectiveness of the proposed method. (ii) Comparing deep neural network-based methods, CADA outperforms 8.31% on *F*1, 5.29% on R, and 10.29% on *P*, because BERT-based methods (i.e. PLMs) have a powerful transformer structure and is pre-trained on large-scale corpora. (iii) Comparing PLMs in the general domain, our method outperforms 1.19% on *F*1, 2.24% on *R*, and 0.22% on *P*, further proving the effectiveness of our method. The reason is that the performance improvement here is mainly attributed to the superiority of the CADA framework. (iv) Comparing PLMs in the biomedical domain, our method still improves 0.18% on *F*1, 2.40% on *R*, and 0.28% on *P*. It shows that the superior performance of our framework can compensate for the gap caused by the domain corpus to some extent.

**Table 4.** Main experiment results on the test set of English ChemProt.

Method	ChemProt		
	*P* (%)	*R* (%)	*F*1 (%)
Recognition network	56.10	67.84	61.41
SVM + I-ANN	59.05	67.76	63.10
Majority voting	72.66	57.35	64.10
SPINN	74.8	56.0	64.1
GA-BGRU	65.44	64.84	65.14
CPP-BiLSTM	70.6	61.8	65.9
DS-LSTM	67.04	72.01	69.44
CasRel(BERT encoder)	52.7	55.6	54.1
NovelTagging(BERT encoder)	63.7	47.8	54.6
MRC4BioER(BIO tagging)	65.0	62.7	63.8
MRC4BioER	70.9	61.7	66.0
GPLinker	68.57	68.86	68.71
BERT+Gaussian	77.08	76.06	76.56
BERT+balance bagging	69.0	71.0	70.0
BERT+DARE	79.0	68.0	73.0
NCBI BERT	74.5	70.6	72.5
BioBERT	77.02	75.90	76.46
PubMedBERT	-	-	77.24
BioLinkBERT	-	-	77.57
CADA(our)	77.30	**78.30**	**77.75**

#### 3.2.2 Results of the subtasks experiment

We continue to explore the performance of CADA on various BioRE subtasks, i.e. entity pair extraction and RC. Table 5 shows that CADA gains around 2% performance improvement over SoTA baseline on all subtasks on the CMeIE validation set. Besides, on the ChemProt test set, our method improves by 7.40% and 4.45% on entity pair extraction and RC subtasks, respectively. These encouraging results once again verify our effectiveness. We conclude that introducing the DA can effectively enhance the association of entity pairs and relations within triplets.

**Table 5.** Results on triplet elements.^a^

Method	Element	CMeIE			ChemProt		
		*P* (%)	*R* (%)	*F*1 (%)	*P* (%)	*R* (%)	*F*1 (%)
GPLinker	(s,o)	79.33	65.43	71.71	72.67	72.75	72.71
	*r*	78.99	67.38	72.73	79.38	77.08	78.21
	(s,r,o)	66.39	56.70	61.17	68.57	68.86	68.71
CADA(our)	(s,o)	77.71	69.46	**73.35**	79.46	80.78	**80.11**
	*r*	77.88	70.88	**74.22**	83.54	81.81	**82.66**
	(s,r,o)	66.51	60.60	**63.43**	77.26	78.26	**77.75**

a (s,o) denotes entity pair and *r* denotes relation.

### 3.3 Detailed results on complex scenarios

Chinese BioRE is more challenging than English because Chinese biomedical texts have no clear word separator (Cai et al. 2021). Therefore, subsequent performance experiments and analyses are performed on Chinese CMeIE. In addition, as the CMeIE test labels are not released, experiments in this section are conducted on validation set.

#### 3.3.1 Results of various overlapping patterns

**Figure 8.**
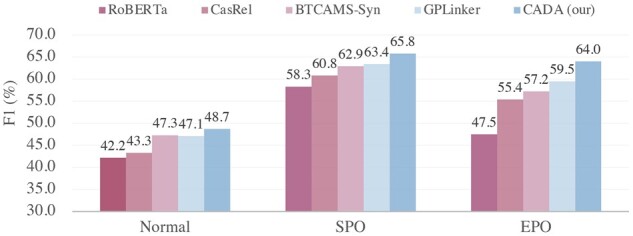
*F*1 of various overlapping patterns.

We further explore complex samples containing various overlapping patterns on the CMeIE to verify the ability of the CADA. For the three experiments, we choose to compare them with the SoTA methods of published papers. Figure 8 shows that our CADA outperforms all the baselines in SPO and EPO patterns, and CADA exhibits excellent performance in dealing with overlapping problems. It is because the DA handles complex overlap problems in the biomedical domain and the data imbalance between entity pairs and relations, i.e. triplet overlap problem.

#### 3.3.2 Results of multiple triplets in a sample

When addressing the issues of multiple triplets, an *N*≥5 sample may contain SEO and EPO patterns simultaneously, which brings multiple challenges to extraction methods. Table 6 shows that CADA outperforms the baseline and exhibits encouraging capabilities in multiple triples. Attributable to CADA having the ability of DA, SO2R, and R2SO match triplets in two directions.

**Table 6.** *F*1 of multiple triplets in a sample.

Method	Different numbers of triplets				
	*N* = 1	*N* = 2	*N* = 3	*N* = 4	*N* ≥5
RoBERTa	43.0	48.4	54	56.8	62.6
CasRel	44.4	52.8	56.8	57.8	64.0
BTCAMS-Syn	47.8	**55.8**	56.9	58.1	66.8
GPLinker	47.3	53.7	58.6	61.2	67.4
CADA(our)	**50.0**	55.5	**61.8**	**64.4**	**70.0**

#### 3.3.3 Results of cross-sentence triplets

**Figure 9.**
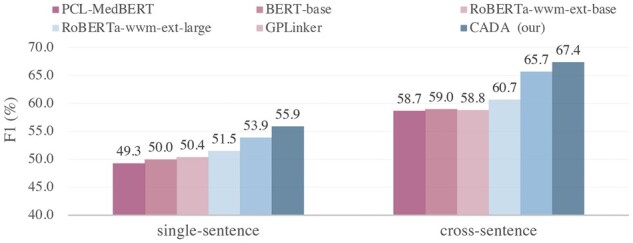
*F*1 of different triplets distance.

We further verified the ability of CADA in the cross-sentence triplets scenario. The results are displayed in Fig. 9. Among them, CADA also exceeds the baseline in both single sentence and cross sentence, which shows that CADA reduce the damage caused by the distance of triplet elements during the extraction process.

### 3.4 Detailed analysis

In this section, we aim to detail an ablation analysis of our method. Besides, we study the impact of various prompts and thresholds, respectively. Eventually, we present a case analysis of the CADA.

#### 3.4.1 Ablation study

We propose a novel framework for BioRE with two additional parts over traditional pipeline methods: DA (i.e. DL and matching modules) and CA. Therefore, we design four sets of ablation experiments better to illustrate the contribution of the additional modules in CADA to model performance. The results are presented in Table 7. From the table, we conclude that: (i) for pipeline-based models, compared with “ER+RC,” the “ER+RC+DA” achieves better results in both the validation set and test set of the CMeIE and ChemProt. In particular, the “ER+RC+DA” increases *F*1 by 1.60% in the CMeIE validation set, and increases *F*1 by 1.76% in the ChemProt test set. For joint-based models, compared “joint+DA” with “joint,” the former achieves better results in four sets. Especially, the “joint+DA” increases *F*1 by 1.78% and 3.63% in the validation set of both CMeIE and ChemProt. This is because introducing DA in pipeline methods can play the role of checking, extracting the reverse thinking of entity pairs through relation information, and enhancing the connection between entity pairs and relations. (ii) The “ER+RC+DA+CA” are better than the “ER+RC+DA” on both pipeline-based and joint-based methods. The reason is that designing the CA algorithm can reasonably optimize the data flow propagation of CADA. As a result, our method effectively reduces the effect of pipeline-based process error propagation and intuitively reduces redundant entity extraction. (iii) The two additional parts have enhancements for both pipeline methods and joint methods, indicating that our method is not limited by the method types. We observe that with the superposition of the additional parts, precision score keeps increasing in all datasets and models. In other words, our method corrects errors generated by the previous steps, e.g. entity pairs without relations may be incorrectly assigned relations, shown in Section 3.4.5.

**Table 7.** Ablation study of our model.

Model		Method	CMeIE			ChemProt		
			*P* (%)	*R* (%)	*F*1 (%)	*P* (%)	*R* (%)	*F*1 (%)
Validation set								

Pipeline	CADA	ER+RC	59.91	62.99	61.41(−1.60)	72.18	81.44	76.53(−1.40)
		ER+RC+DA	64.96	61.26	63.01(−0.42)	75.76	79.94	77.80(−0.13)
		ER+RC+DA+CA	66.51	60.60	**63.43**	76.14	79.82	**77.93**
Joint	GPLinker	joint	66.39	56.70	61.17(−1.78)	70.57	68.49	69.52(−3.63)
		joint+DA	71.51	55.72	62.64(−0.31)	79.71	66.87	72.73(−0.42)
		joint+DA+CA	72.83	55.43	**62.95**	81.62	66.27	**73.15**

Test set								

Pipeline	CADA	ER+RC	61.31	62.52	61.91(−1.22)	72.41	79.95	75.99(−1.76)
		ER+RC+DA	63.95	61.92	62.92(−0.21)	77.38	77.77	77.58(−0.17)
		ER+RC+DA+CA	65.44	60.98	**63.13**	77.26	78.26	**77.75**
Joint	GPLinker	joint	67.07	55.84	60.95(−1.07)	68.57	68.86	68.71(−1.24)
		joint+DA	65.10	59.21	62.02(−0.07)	75.11	65.17	69.79(−0.16)
		joint+DA+CA	69.49	56.12	**62.09**	73.50	66.74	**69.95**

#### 3.4.2 The impact of prompts

To extract the entity pair (s,o) when the relation *r* is known, we use prompt technique to concatenate the *r* with text *C* as the input. Note that the design of prompt template is not unique, and comparative experiments are done to demonstrate that the current template works better. Thus, we design five prompt templates, as shown in Table 8. Among them, [/s1], [/s2], and [/s3] denote separation tokens, $\left\{r_{\mathrm{type}}\right\}$, $\left\{s_{\mathrm{type}}\right\}$, and $\left\{o_{\mathrm{type}}\right\}$ denote relation type, subject type, and object type, respectively.

**Table 8.** Results on DL module with different prompts.

Prompt		*P* (%)	*R* (%)	*F*1 (%)
1	[/s1]$\left\{r_{\mathrm{type}}\right\}$	65.183	61.091	63.031
2	[/s1]$\left\{s_{\mathrm{type}}\right\}$[/s2]$\left\{o_{\mathrm{type}}\right\}$	65.183	61.091	63.071
3	[/s1]$\left\{r_{\mathrm{type}}\right\}$[/s2]$\left\{s_{\mathrm{type}}\right\}$	64.900	61.158	62.973
4	[/s1]$\left\{r_{\mathrm{type}}\right\}$[/s2]$\left\{o_{\mathrm{type}}\right\}$	65.173	61.106	63.074
5	[/s1]$\left\{r_{\mathrm{type}}\right\}$[/s2]$\left\{s_{\mathrm{type}}\right\}$[/s3]$\left\{o_{\mathrm{type}}\right\}$	65.068	61.210	**63.080**

Specifically, to verify the difference between the five prompt templates, we use the consistent results of the ER and RC modules on the CMeIE validation set, and the parameters of the DL module keep the same without the CA tuning algorithm. Comparing the results of the DL module, it can be seen from Table 8 that the No. 5 prompt template has the best effect. The one reason may be that there are 53 schemas in the CMeIE, including 10 synonymous relations and 43 other relations, where the relation types of the synonymous relations are the same, but the *s* and *o* entity types are different. So No.1 prompt template, which contains only the relation types, does not work well. The another reason may be that the subject entity types of CMeIE are all diseases, only introducing subject types is equivalent to introducing noise. Thus, No.3 prompt templates, which contain the subject entity types, perform worse than No.1 prompt template. No.2, No.4, and No.5 prompt templates have little difference in performance, and finally we choose No.5 based on the best performance.

#### 3.4.3 The impact of various thresholds

To explore the effectiveness of the CA algorithm, we design experiments from two aspects to aim to study the impact of various module thresholds on framework performance. First, we show the performance variation of each module under different threshold settings to illustrate that each selected threshold impacts the overall framework performance. Second, the effectiveness of our threshold combination strategy is validated by showing the variation in results for interval sampling of thresholds. From Table 9, we observe that: (i) all four selected thresholds modulate the performance of the entire framework, with the matching threshold having the most obvious impact. (ii) With the CA algorithm, we have the ability to continue improving the performance of the framework.

**Table 9.** Results on different module with different thresholds.

Threshold		*P* (%)	*R* (%)	*F*1 (%)
ER module	0.50	64.799	61.462	63.087
	0.45	64.737	61.499	63.077
	0.40	64.307	61.759	63.007
	0.35	64.055	**61.996**	63.009
RC module	0.40	64.330	61.848	63.065
	0.30	64.239	61.989	63.094
	0.20	64.051	62.093	63.057
	0.10	63.819	**62.204**	63.001
DL module	0.45	65.050	62.217	63.075
	0.50	65.068	62.210	63.080
	0.55	64.386	61.863	63.099
	0.60	64.442	61.840	**63.105**
Matching module	0.80	63.137	62.256	62.693
	0.90	63.951	61.915	62.916
	0.95	64.572	61.529	63.013
	0.98	65.438	60.980	**63.130**

#### 3.4.4 The impact of random seeds

As shown in Table 10, we conduct experiments with five different random seeds, and the results and their improvements are rather robust. We get an averaged performance of 63.14 ± 0.03/77.76 ± 0.2 on CMeIE/ChemProt with five runs in total, which outperforms the previous SoTA methods.

**Table 10.** Results on CADA with different random seeds.^a^

Seed	CMeIE			ChemProt		
	*P* (%)	*R* (%)	*F*1 (%)	*P* (%)	*R* (%)	*F*1 (%)
41	65.53	60.96	63.16	77.36	78.23	77.79
42	66.34	60.24	63.14	76.55	78.77	77.65
43	65.75	60.71	63.13	77.84	77.74	77.80
44	65.37	61.03	63.12	76.71	78.98	77.83
45	65.59	60.83	63.13	76.53	78.95	77.72
avg	65.72${}_{\pm 0.8}$	60.75${}_{\pm 0.6}$	**63.14** ${}_{\pm 0.03}$	77.00${}_{\pm 1.2}$	78.53${}_{\pm 1.1}$	**77.76** ${}_{\pm 0.2}$

a We report average scores with standard deviations as subscripts.

#### 3.4.5 Case study

**Figure 10.**
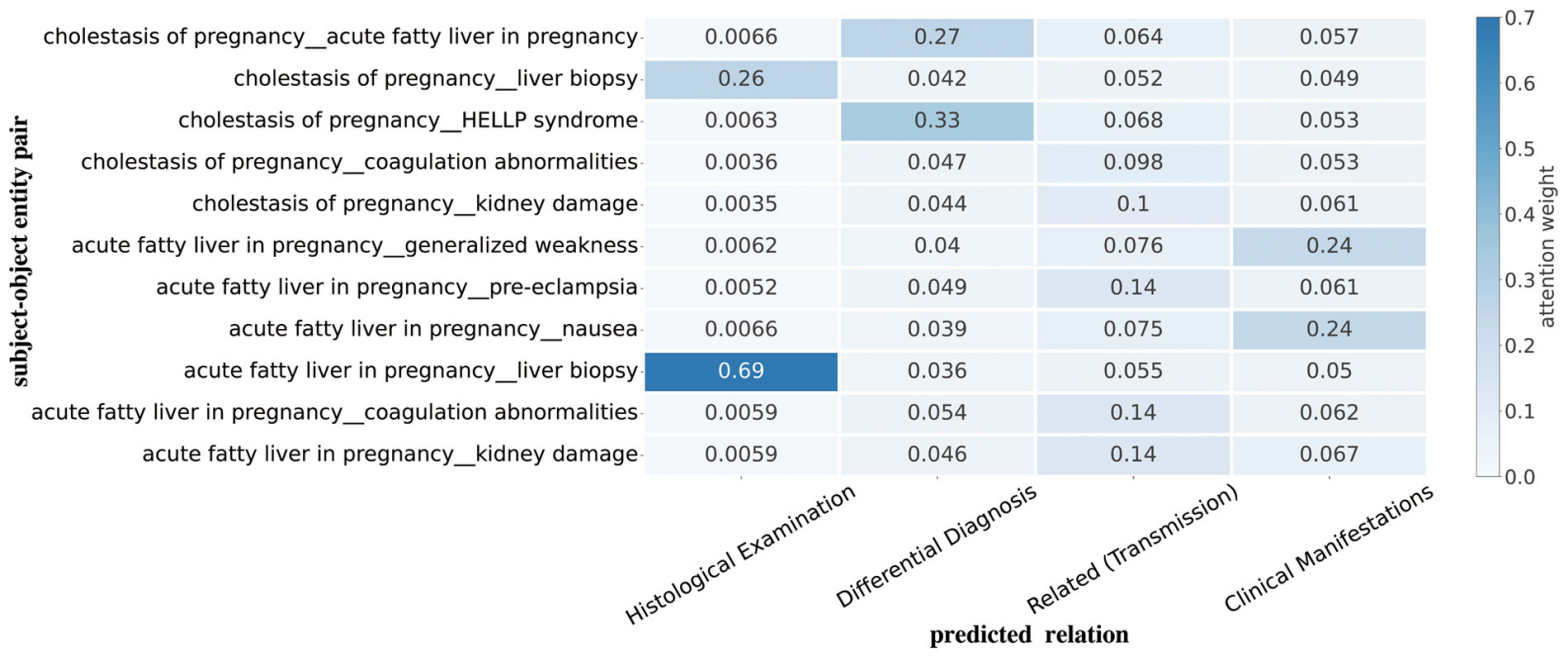
Visualization of the attention weight between subject–object entity pair and relation on ER and RC modules.

**Figure 11.**
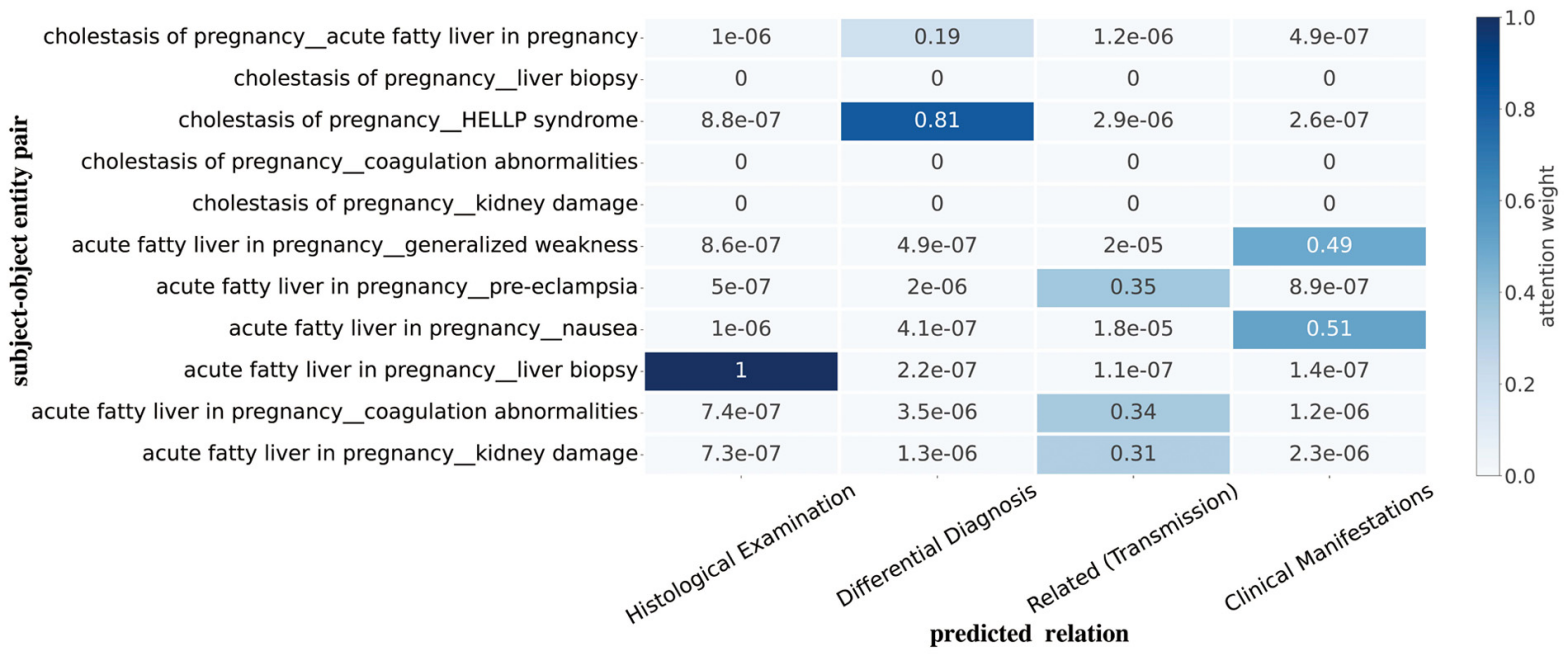
Visualization of the attention weight between subject–object entity pair and relation on CADA.

To show the effectiveness of our proposed DA and CA algorithm, we visualize the attention weights between subject–object entity pair and relation. In Fig. 10, we present the attention weight results for the ER and RC modules. Specifically, we compute the attention weight between the subject–object entity pair and relation using the output layer of the RC model. To aggregate attention scores, we calculate the average of multiple attention heads. Figure 11 shows the result of CADA. Comparing the above two diagrams, we observe that CADA enhances the connections between entity pairs and relations within the target triplets and excludes the wrong triplets.

**Figure 12.**
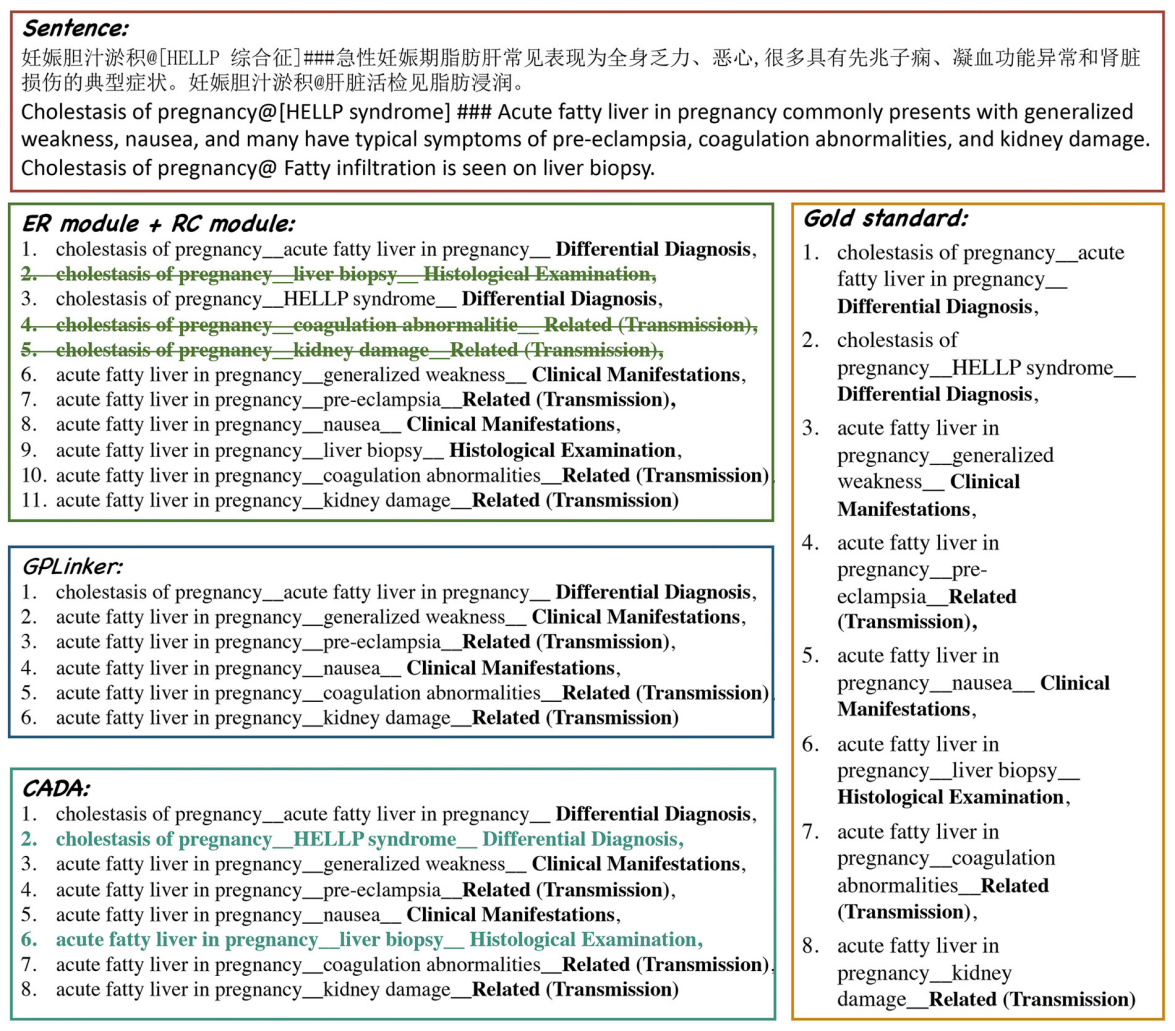
Example prediction results on the CMeIE. The serial number denotes the results of the relation triplet extracted by each method, in the form: subject_object_relation. Among them, the black denotes the correct answer, the green strikethrough (i.e. crossed-out row) denotes the wrong answer, and the blue denotes the correct answer that our method can extract but GPLinker cannot.

Besides, to analyze the advantages of our proposed method, we compare the prediction results of ER module + RC module (i.e. traditional pipeline method) and GPLinker. The example of prediction results is shown in Fig. 12. The case involves eight relations. The “ER module + RC module” method employs a span-based ER model and a classifier to extract these triplets, resulting in redundant RE. GPLinker extracts the entities and relations by joint learning model, whose result is that the prediction misses two triplets. The results of CADA are correct, correcting the previous error with the DL and matching modules and predicting the two triplets missed by GPLinker.

#### 3.4.6 Error analysis

We further analyze the errors and present the distribution of five errors in Table 11. For the CMeIE, a total of 3585 samples are used for error analysis. The number of samples that GPLinker do wrong but our method do right is 312, accounting for 8.70%. For the ChemProt, a total of 1392 samples are used. The number of samples that BioLinkerBERT do wrong but our method do right is 179, accounting for 12.86%.

**Table 11.** Distribution of five RE errors on CMeIE and ChemProt.

Type	CMeIE		ChemProt	
	Samples	Proportion (%)	Samples	Proportion (%)
Entity span error	184	49.60	127	64.14
Subject	72	36.55	75	49.17
Object	125	63.45	84	52.83
Entity type error	20	5.39	0	0
Relation error	44	11.86	19	8.26
Triplet not found	100	26.95	52	22.61
Triplet redundancy	23	6.2	0	0

#### 3.4.7 Robustness analysis

We further analyze the robustness of our method on BioRE task. Due to CADA achieves SoTA performance on ChemProt from BioCreative VI competition, we select to use the DrugProt from BioCreative VII competition. Now that the competition has ended, it is worth noting that released DrugProt dataset only consists of a training set and a validation set. Our findings are presented in Table 12, CADA outperforms all baselines in terms of *P* and *F*1, reaching 83.33% and 82.78%, respectively, which proves the robustness of the CADA.

**Table 12.** Experimental results on the DrugProt dataset.

Method	DrugProt (valid)		
	*P* (%)	*R* (%)	*F*1 (%)
CU-UD	77.70	77.80	77.70
TTI-COIN	78.00	75.20	76.60
NLM-NCBI	81.10	82.50	81.90
Humboldt	80.40	79.70	80.10
CADA	**83.33**	82.24	**82.78**

## 4 Conclusion

In this article, we propose a novel co-adaptive framework with duality-aware for BioRE. Unlike previous methods, the CADA utilizes the duality mechanism to transform the BioRE task into dual tasks, achieving entity boundary identification twice and aiming to correct the errors in the early steps. Specifically, our proposed CADA first employs a span-based entity model to identify subject–object entity pairs. Then, we train a classifier with entity marker techniques for RC. Besides, we design the duality module with prompt learning techniques to extract the corresponding entity pairs based on relation type. Finally, match the RC results and DL results to the final result. In addition, a co-adaptive training strategy and a CA algorithm are designed to further improve CADA performance. Compared to existing methods, extensive experimental results on the CMeIE and ChemProt datasets show that CADA extract the entities and relations more accurately. CADA also mitigates to some extent the domain-specific problems of BioRE, including overlapping relations, multiple triplets in a single sentence, and cross-sentence triplets.
